# The Immune Memory Response of In Vitro-Polarised Th1, Th2, and Th17 Cells in the Face of Ovalbumin-Transgenic *Leishmania major* in a Mouse Model

**DOI:** 10.3390/ijms25168753

**Published:** 2024-08-11

**Authors:** Mebrahtu G. Tedla, Musammat F. Nahar, Alison L. Every, Jean-Pierre Y. Scheerlinck

**Affiliations:** 1 Department of Pediatrics, School of Medicine, University of Missouri-Columbia, Columbia, MO 65211, USA; 2 Department of Health Science and Community, Swinburne University of Technology, Hawthorn, VIC 3122, Australia; 3 Australian Academy of Technological Sciences and Engineering, Forrest, ACT 2603, Australia; 4 Centre for Animal Biotechnology, Faculty of Veterinary and Agricultural Sciences, The University of Melbourne, Parkville, VIC 3010, Australia; j.scheerlinck@unimelb.edu.au

**Keywords:** immune memory, in vivo, in vitro, OVA, immune protection, *Leishmania major*, T helper cells

## Abstract

Th1 and Th2 cytokines determine the outcome of *Leishmania major* infection and immune protection depends mainly on memory T cells induced during vaccination. This largely hinges on the nature and type of memory T cells produced. In this study, transgenic *Leishmania major* strains expressing membrane-associated ovalbumin (mOVA) and soluble ovalbumin (sOVA) were used as a model to study whether fully differentiated Th1/Th2 and Th17 cells can recall immune memory and tolerate pathogen manipulation. Naïve OT-II T cells were polarised in vitro into Th1/Th2 cells, and these cells were transferred adoptively into recipient mice. Following the transferral of the memory cells, the recipient mice were challenged with OVA transgenic *Leishmania major* and a wild-type parasite was used a control. The in vitro-polarised T helper cells continued to produce the same cytokine signatures after being challenged by both forms of OVA-expressing *Leishmania major* parasites in vivo. This suggests that antigen-experienced cells remain the same or unaltered in the face of OVA-transgenic *Leishmania major.* Such ability of these antigen-experienced cells to remain resilient to manipulation by the parasite signifies that vaccines might be able to produce immune memory responses and defend against parasitic immune manipulation in order to protect the host from infection.

## 1. Introduction

*Leishmania major* causes a wide spectrum of disease outcomes, ranging from asymptomatic to fatal infections. This is not only due to variations in the pathogenicity of the parasitic strains but is also due to differences in the susceptibility of different hosts [1]. Early immune responses during *Leishmania* infection determine the prognosis of the disease, with it being either acute (self-clearing) or chronic. To investigate these, various experimental studies have been conducted in mouse strains. Following *L. major* infection, some mouse strains develop CD4+ Th1 cell-mediated resistance, whereas other strains show a CD4+ Th2-mediated response and are very susceptible to infection [2]. For example, amongst mouse strains, C57BL/6 mice can resolve infections and produce long-lasting immunity, while BALB/c mice are unable to control infections, leading to disease progression in most cases. The ability of C57BL/6 mice to control infection is linked to their production of Th1 cytokines [3]. The expression of IFN-γ has been shown to be associated with resolving infection in human [4,5] and murine *Leishmaniasis* [6]. Macrophage activation also plays a crucial role and IFN-γ is required as a macrophage-activating factor during elimination of *Leishmania* [7,8]. IL-4 is also important in macrophage activation [9] and in the upregulation of CR3, a macrophage receptor for *Leishmania* [10,11]. Acquired resistance to *L. major* infection relies on the activation of CD4+ T cells, which leads to the production of large amounts of IFN-γ, and this induces parasite killing by macrophages following nitric oxide release [12,13,14]. Though the killing of the parasite is mainly orchestrated by IFN-γ through direct macrophage activation [6,7,8], the direct inhibition of IL-4 [15,16] and the reduced clonal expansion of Th2 cells caused by IFN-γ [17] are also important. In addition, direct mechanisms allowing IL-4 to inhibit IFN-γ activity have been identified, including the direct inhibition of macrophage activation to eliminate intracellular amastigotes [18]. Although CD4+ T cells produce IL-10, which is important in promoting parasite survival, how such responses develop from precursors during infection is not known [19]. Therefore, one can conclude that both Th1 and Th2 cytokines contribute in opposing ways to the pathogenic outcome of *Leishmania* infection [20].

Despite the promising progress in developing vaccines against *Leishmania*, complete immune protection is not easy to achieve due to the lack of persistent parasites and sterile immunity. This is due to the fact that parasite antigens are not effective in inducing long-term immune memory. Another challenge in achieving vaccine-mediated immune protection is the lack of immunodominant antigens that can be recognised by CD4+ T cells [2]. Immune memory produced after secondary infection with *L. major* is important in conferring and maintaining protection through central memory CD4+ T cells. Indeed, these memory T cells become tissue-homing effector cells that can induce protection against infection. Therefore, immune protection against *L. major* could be mediated through memory T cells induced during vaccination and this largely depends on the nature and type of memory T cells produced [21]. During pre-existing immunity, the secondary site of parasite infection leads to IFN-γ- and monocyte-mediated parasite killing [22]. Studies in resistant C57Bl/6 mice have shown pre-existing effector CD4 T cells (CD44+CD62L−T-bet+Ly6C+ effector (T_EFF_)) generated by ongoing chronic infection and that these are short-lived if there is no infection [23]. Indeed, if the immune effector cells are of the Th1 type and they can withstand any manipulation by the parasite, they may induce protection. If, on the other hand, Th2 cells are induced or the Th1 immune responses are manipulated by the parasite to produce Th2 cytokines during the recall response, it is possible that the disease might be exacerbated. Therefore, it is essential to establish whether the induced immune memory is resilient to manipulation by the parasite.

OT-II CD4+ T cells are known to express transgenic OVA-specific αβ-TCRs, and the use of OVA as a model antigen is ideal as this effectively excludes the intrinsic biases possibly associated with parasite-derived antigens. Therefore, the use of OVA as a neutral antigen allows us to study the immune responses induced by a parasite in vivo and in vitro. The OVA antigen being expressed by the parasite ensures that it is processed and presented by the same antigen-presenting cells as those in the parasite, and hence the parasite-derived immunomodulatory molecules, if present, are in the in the ideal microenvironment to influence the immune response against the expressed model antigen.

In this study, an OVA-transgenic *L. major* parasite expressing OVA in either a membrane-bound or a soluble form was used as a tool to address aspects of CD4+ T cell activation, immune memory resilience, and pathogen-mediated manipulation in an OT-II TCR-transgenic mouse model.

## 2. Results

### 2.1. Cloning of sOVA and mOVA in Leishmania Expression Vector (pIR1SAT)

**Figure 1:**
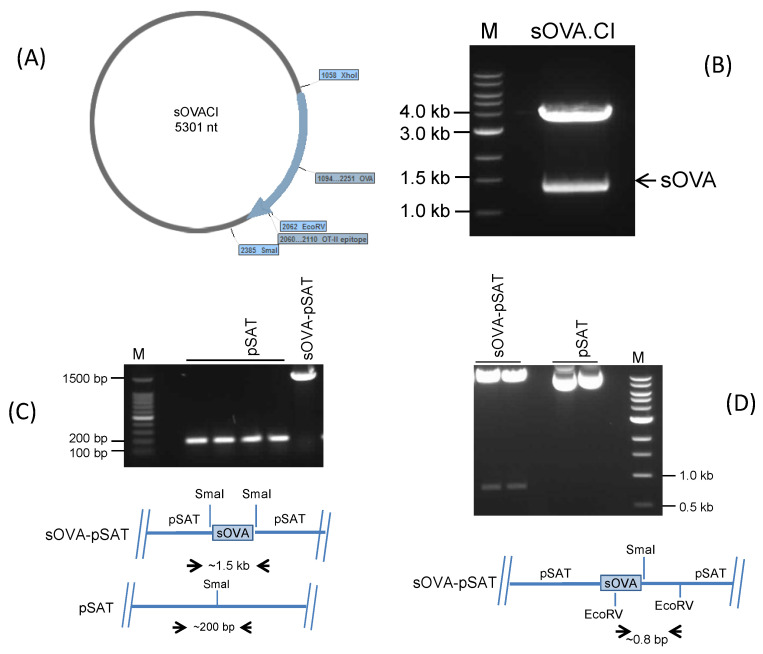
Restriction digestion of the sOVA.CI plasmid and cloning of the sOVA fragment into the pSAT vector. (**A**) Restrictive digestion was used to remove the sOVA fragment from the sOVA.CI plasmid. (**B**) sOVA.CI was digested by XhoI and SmaI.Cl. The arrow denotes a fragment that was later gel-purified and corresponds to the DNA responsible for encoding sOVA. sOVA, OVA that has been secreted; M, molecular weight marker; sOVA.CI, plasmid carrying sOVA. The sOVA fragment was cloned into the pSAT vector. (**C**) Following colony PCR, using forward and reverse primers created on the pSAT vector backbone, gel electrophoresis was conducted, showing amplification of the sOVA-pSAT-positive clone (2 kb). (**D**) Due to the presence of one EcoRV site on the sOVA insert and one on the pSAT vector, restriction digestion of the recombinant sOVA-pSAT plasmid with EcoRV produced a 0.8 kb fragment of the positive clones with correct orientation. M, molecular weight marker; pSAT, only the pSAT vector; sOVA, secreted OVA; sOVA-pSAT, sOVA in the pSAT vector.

Since transfection with the targeting region of the plasmid permits recombination of the desired gene into the parasite chromosome and stable expression, the *Leishmania* expression vector, pIR1SAT, was used to express OVA (SERPINB14). As a result, the sOVA.CI plasmid was digested using the restriction enzymes XhoI and SmaI to produce a 1158 bp fragment that matched OVA and contained the N-terminal signal sequence for secretion. This fragment was subsequently gel-purified using a gel purification kit from Promega in accordance with the manufacturer’s instructions (Figure 1A,B). The Klenow enzyme treatment blunted the ends of the purified sOVA fragment. To construct the vector pSAT-sOVA, the *Leishmania* expression vector pIR1SAT, also known as pSAT, was digested with SmaI and the gene encoding the sOVA-expressing fragment gene was introduced using conventional ligation procedures. The ligated DNA was utilised to transform *E. coli* TOP10, and colony PCR was used to check for clones having the right insert. In order to find clones with the insert in the right orientation, plasmid DNA was extracted from positive clones and screened using EcoRV digestion (Figure 1C,D). The sequence encoding the N-terminal 139 amino acids of sOVA were replaced with the sequence encoding the *Leishmania* transferrin receptor in the construct responsible for membrane localisation, which was used to express membrane-associated OVA (mOVA).

### 2.2. Expression of OVA by Transfected L. major

**Figure 2:**
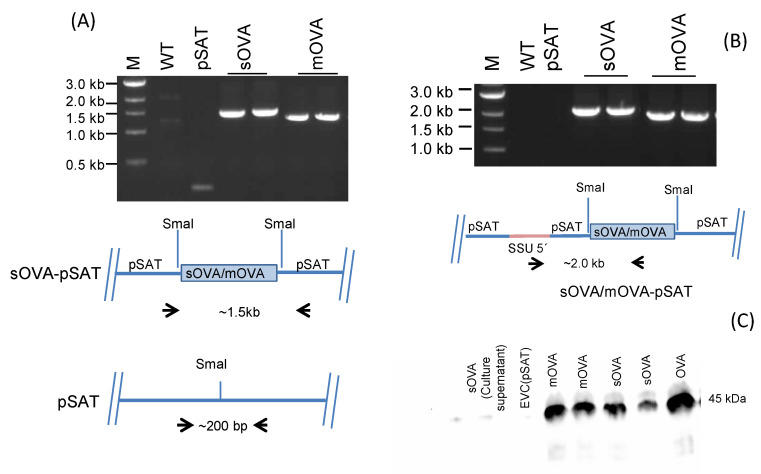
Transfection of *L. major* with sOVA- and mOVA-encoding DNA sequences containing recombinant pSAT. (**A**) OVA was detected in the genomic DNA that was taken from the transfected *L. major*. Both forward and reverse primers were created using the pSAT vector backbone to conduct the PCR experiment. (**B**) Using primers based on the *Leishmanial* SSU region (forward) and the middle of the OVA fragment (reverse), the integration of the OVA transgene into the *Leishmanial* chromosome was verified. M stands for molecular weight marker, WT for wild-type, pSAT for pSAT vector only, SSU 5′ for small subunit of the *Leishmanial* chromosome 18s rRNA, sOVA-pSAT for sOVA in the pSAT vector, and mOVA-pSAT for mOVA in the pSAT vector. (**C**) For the confirmation of OVA expression, whole parasite lysates were confirmed by using SDS-PAGE followed by subjecting them to anti-OVA immunoblotting. A total of 2 × 10^7^ log-phase promastigotes were lysed to detect protein expression. Both membrane-associated OVA and soluble OVA were detected in the transformed parasites. The OVA protein and a parasite empty-vector control were used as positive and negative controls, respectively. The first two lanes show culture supernatants of log-phase *L. major* promastigotes.

To create parasites (and control lines) that stably produce the OVA protein, the *L. major* parasites were transfected with the pSAT-mOVA, pSAT-sOVA, and pSAT plasmids. In order to achieve this, these plasmids were digested with SwaI to produce fragments comprising the 5′ and 3′ targeting areas (homologous to 18s rRNA SSU) and containing the DNA encoding either mOVA, sOVA, or the pSAT plasmid solely, respectively. These pieces were electroporated into the parasite chromosome and then replaced one 18s rRNA SSU fragment. By using PCR amplification, it was confirmed that the recombinant DNA had been successfully transfected into the parasite (Figure 2A). The vector backbone was amplified via PCR using only the pSAT transfection, which resulted in a 200 bp fragment, as opposed to the expected 1.5 kb and 1.3 kb pieces from the sOVA- and mOVA-containing pSAT constructs. PCR amplification of the inserted DNA using primers flanking the region of insertion, confirming integration into the right location of the genome, further established the OVA-expressing parasite created by the integration of the gene into the parasite chromosome (Figure 2B). The expected 2 kb and 1.8 kb fragments for pSAT-sOVA and pSAT-mOVA, respectively, were produced by the PCR process. *L. major* pSAT (only pSAT DNA transfected), *L. major* pSAT-sOVA (pSAT-sOVA encoding DNA transfected), and *L. major* pSAT-mOVA (pSAT-mOVA expressing DNA transfected) were the three parasite strains that were produced.

The transfection of *L. major* parasites resulted in the successful expression of two forms of the OVA protein. The first form of OVA was secreted OVA (sOVA), and the second form of OVA was membrane-associated OVA (mOVA), which is membrane-anchored via a hydrophobic transmembrane sequence. Following transfection of the mid-log-phase promastigotes with the pSAT-mOVA plasmid, the pSAT-sOVA plasmid, or the empty-vector control, i.e., the pSAT plasmid, the successful expression of the OVA protein was confirmed using the Western blot method (Figure 2C). To confirm the expression of the OVA protein using Western blotting, a pool of approximately 2 × 10^6^ promastigotes was cultured until they reached into the mid-log phase and a total of 2 × 10^7^ parasites were lysed using the heat-shock method (56 °C for 10 min), and the protein was subjected to SDS-PAGE, transferred to a nitrocellulose membrane, and detected using OVA-specific antibodies. The supernatant was also assayed separately, but no band was detected (Figure 2C). Promastigotes transfected with pSAT-mOVA and pSAT-sOVA expressed the protein having a band size of 45 kDa (Figure 2C), which is the same size as the chicken OVA protein. The empty-vector control here, referred to as the pSAT plasmid, was used as a negative control and showed no expression of OVA by the parasite.

### 2.3. OT-II CD4^+^ T Cell Proliferation by OVA-Expressing L. major

**Figure 3:**
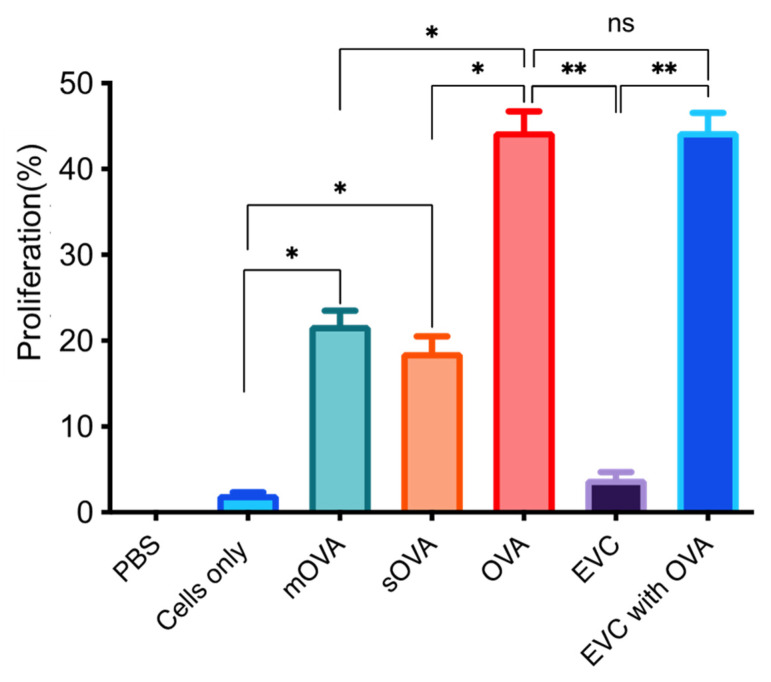
The proliferation of OT-II T cells following stimulation with OVA-expressing *L. major* parasites in vitro. A single-cell suspension was prepared from the splenic, maxillary, axillary, and inguinal lymph nodes of OT-II mice for antigenic stimulation of either the whole fraction or the non-purified cells. Cells were labelled with cell-division-tracking dye, CFSE (0.5 μΜ), to a total of 2 × 10^5^ cells per well in a 96-well plate, with a total of 8 wells for each parasite strain, and after 4 days of incubation, the pool of cells were analysed for proliferation using PE-anti-mouse CD4 marker (Bio Legend). Proliferation was analysed using flow cytometry relaying on the CFSE dye being diluted following each cell division. EVC stands for empty-vector control, mOVA stands for membrane-associated OVA, sOVA stands for soluble OVA. *, *p* < 0.05; **, *p* < 0.01; ns, not significant.

The in vitro activation of OT-II CD4^+^ T cells after exposing the cells to OVA-expressing parasites was determined following the extraction of these cells from the spleens and lymph nodes of the OT-II mice. The results shown in Figure 3 demonstrate that cells stimulated with mOVA-expressing parasites had a higher proliferation level (21.8%) than those exposed to sOVA-expressing parasites (18.7%). Cells exposed to the OVA peptide (10 μg/mL) and OVA protein (10 μg/mL) showed 61.3% and 44.4% proliferation, respectively. To ascertain whether the presence of *L. major* affects OT-II T cell proliferation, the OVA peptide and OVA protein were mixed with the empty-vector control and the responses were compared to the proliferation in the absence of lysate. The results show that the level of proliferation, 61.5% vs. 61.3% for the OVA peptide and 44.2% vs. 44.4%, for the OVA protein, was not affected by the presence of the parasite (Figure 3). Some marginal background proliferation was revealed when cells were exposed to parasites transfected with empty-vector controls and when cells were left unstimulated (3.9% and 2.1% proliferation, respectively; see Figure 3).

### 2.4. Expression of Th Cytokines Following Activation with OVA-Expressing L. major

**Figure 4:**
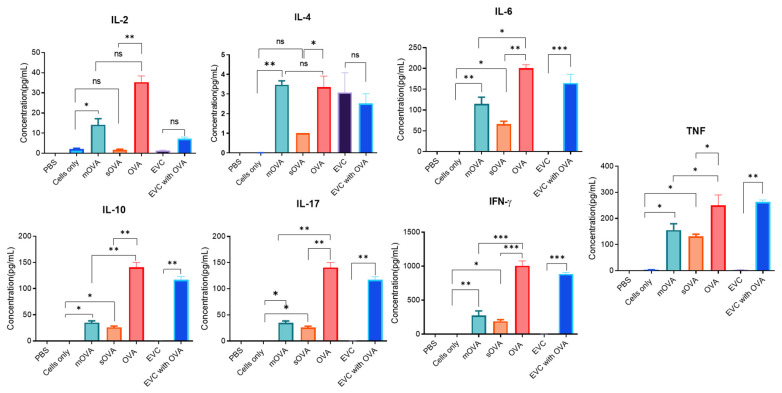
Detection of cytokines released by OT-II T cells stimulated with OVA-expressing parasites. A single-cell suspension was prepared from the splenic, maxillary, axillary, and inguinal lymph nodes of OT-II mice for antigenic stimulation of either the whole fraction or the non-purified cells. A total of 2 × 10^5^ OT-II T cells per well in a 96-well plate were cultured with each parasite strain, and after 4 days of incubation, culture supernatants were taken to analyse the secretion of cytokines using a cytometric bead array. EVC stands for empty-vector control, mOVA stands for membrane-associated OVA, sOVA stands for soluble OVA. *, *p* < 0.05; **, *p* < 0.01; ***, *p* < 0.001; ns, not significant.

The cytokine profiles of OT-II CD4^+^ T cells after exposure to OVA-expressing *L. major* were analysed in the culture supernatants of these cells. A total of seven cytokines representing the three T helper cell lineages, namely Th1, Th2, and Th17, were analysed. The results revealed that a significant amount of IFN-γ, TNF, IL-17, and IL-6 was detected in cells stimulated with mOVA- and sOVA-expressing parasites compared to the cells stimulated with an equal number of parasites transfected with the empty vector (as a control) and the cells left unstimulated (Figure 4). The expression of IL-2, as well as that of IL-10, was significantly higher in cells treated by OVA compared to those treated with mOVA- and sOVA-expressing parasites. In addition, a comparison of the expression levels of all cytokines between the cells stimulated with mOVA and those stimulated with sOVA showed no statistical significance (Figure 4).

### 2.5. OT-II T Cells’ Response to OVA-Expressing L. major

**Figure 5:**
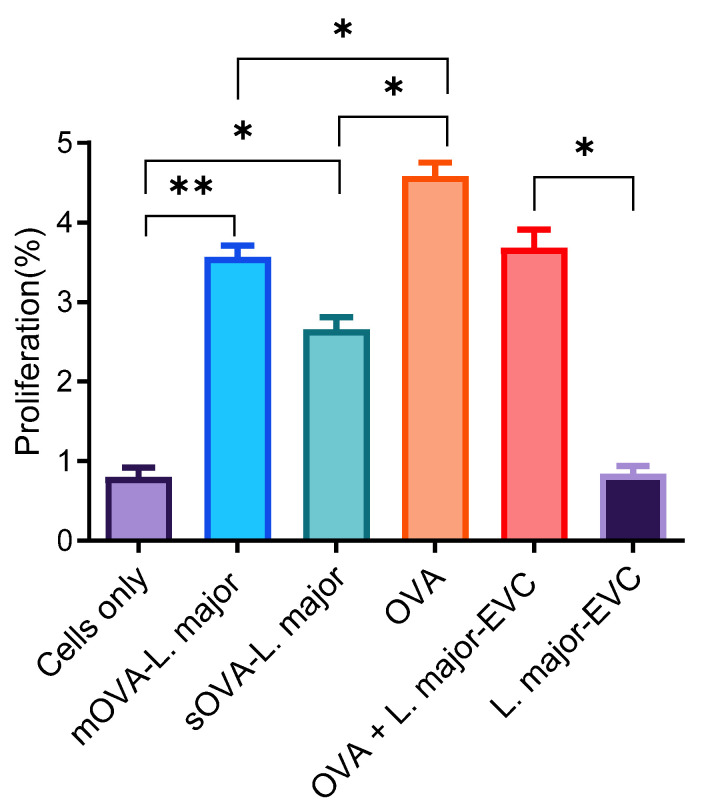
Proliferation rate of recovered OT-II T cells after parasite challenge. A single-cell suspension was prepared from the splenic, maxillary, axillary, and inguinal lymph nodes of OT-II mice for antigenic stimulation of either the whole fraction or the non-purified cells. After the injection of 2 × 10^6^ CFSE-labelled OT-II cells into CD45.1-congenic mice, 5 × 10^6^ OVA-expressing *Leishmania* promastigotes and empty-vector-transformed promastigotes (EVC) were given s.c. to each mouse. Unchallenged OVA- (20 μg/mL) and CFSE-labelled cells were used as positive and negative controls. The time between the first day of cell injection and the challenge with the antigens was 24 h. After the challenge, infection was maintained for a week before the mice were culled for cell recovery and cells were extracted from their spleens and lymph nodes. After a week, the recovered cells were directly analysed. EVC stands for empty-vector control-transfected *Leishmanial* parasites. *, *p* < 0.05; **, *p* < 0.01.

The recognition of OVA expressed by *L. major* promastigotes was analysed following the transferral of 2 × 10^6^ CFSE-labelled OT-II CD4+ T cells into recipient CD45.1-congenic mice. After 24 h, the mice were subcutaneously injected with 5 × 10^6^ mid-log-phase transgenic parasites. Mice were injected with OVA peptide (10 μg) as a positive control. After one week, cells were extracted from the spleens and lymph nodes of the recipient mice and analysed for their ability to proliferate. The results show a relatively higher percentage of proliferated cells recovered from the mice injected with mOVA (3.53%) and sOVA (2.57%) compared to those recovered from mice that received only cells and mice injected with the empty-vector control (0.84% and 0.89%, respectively; Figure 5). The proportion of proliferating cells in mice that received the OVA peptide was 4.54% (Figure 5).

### 2.6. Ex Vivo Response of OT-II T Cells to OVA-Expressing L. major

**Figure 6:**
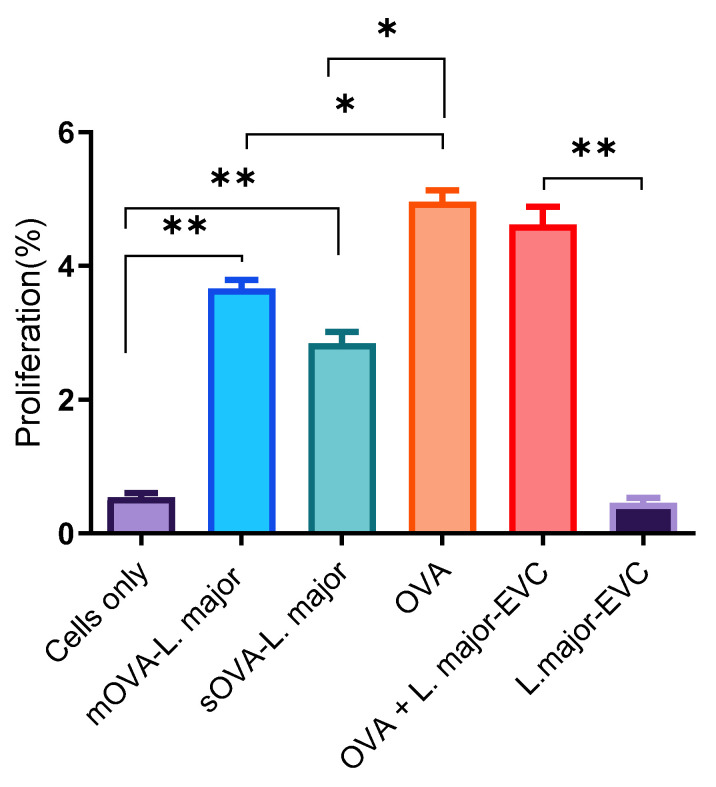
Proliferation rates of recovered cells following ex vivo stimulation. A single-cell suspension was prepared from the splenic, maxillary, axillary, and inguinal lymph nodes of OT-II mice for antigenic stimulation of either the whole fraction or the non-purified cells. After the injection of 2 × 10^6^ CFSE-labelled OT-II cells into the CD45.1-congenic mice, 5 × 10^6^ OVA-expressing *Leishmania* promastigotes and empty-vector-transformed promastigotes (EVC) were given subcutaneously (s.c.) to each mouse. Unchallenged OVA- (10 μg) and CFSE-labelled cells were used as positive and negative controls. The time between the first day of cell injection and the challenge with the antigens was 24 h. After the challenge, infection was maintained for a week before the mice were culled for cell recovery. After a week, cells were extracted from their spleens and lymph nodes and were stimulated ex vivo with each antigen used for the challenge separately for 12 h. EVC stands for empty-vector control-transformed *Leishmanial* parasites. *, *p* < 0.05; **, *p* < 0.01.

As described in the above section, approximately 2 × 10^6^ naïve, unpolarised, CFSE-labelled OT-II T cells were intravenously injected into recipient mice, and after 24 h, each group of mice was challenged by subcutaneous injections with the following antigens: OVA protein (10 μg) as a positive control, 5 × 10^6^ parasites mixed with the same concentration of OVA, 5 × 10^6^ OVA-expressing log-phase promastigotes (mOVA or sOVA), or the same quantity of empty-vector-transformed parasites (EVC); and mice injected with naïve or unpolarised cells were left unchallenged to serve as a negative control. After a week, cells were recovered from spleens and lymph nodes of the recipient mice and further stimulated ex vivo with the same antigens used for the challenge and incubated for four days. After their recovery from the recipient mice, injected OT-II T cells were identified from the recipient cells using anti-mouse CD45.2 marker (the recipient mice are CD45.1). Then, these cells were analysed for their proportion of cell proliferation, and the results, as shown in Figure 6, demonstrate that a significant number of the recovered cells proliferated when stimulated with OVA-expressing *Leishmania* parasites. Only a marginal number of proliferated cells were found in the cells recovered from mice challenged with the EVC treatment alone (0.5%) and in those from mice that remained unchallenged (i.e., no-antigen controls; 0.5%). In contrast, the OT-II T cells recovered from mice challenged with the parasite expressing mOVA, the parasite expressing sOVA, OVA protein, and the OVA protein mixed with the parasite had proliferation rates of 3.7%, 2.8%, 5.0%, and 4.6%, respectively. Using a t-test, the proliferation rate of recovered cells stimulated ex vivo with OVA-expressing *L. major* (parasite expressing both forms of OVA) showed a significantly higher proliferation rate (*p* < 0.05) compared to those recovered from stimulated cells only or empty-vector-transformed parasites (Figure 6). No statistical significance was observed among the recovered cells ex vivo between those stimulated with the mOVA-expressing parasite and those stimulated with the sOVA-expressing parasite (*p* < 0.05, Figure 6).

### 2.7. Cytokine Production of Recovered Cells Following Stimulation with OVA-Expressing L. major

**Figure 7:**
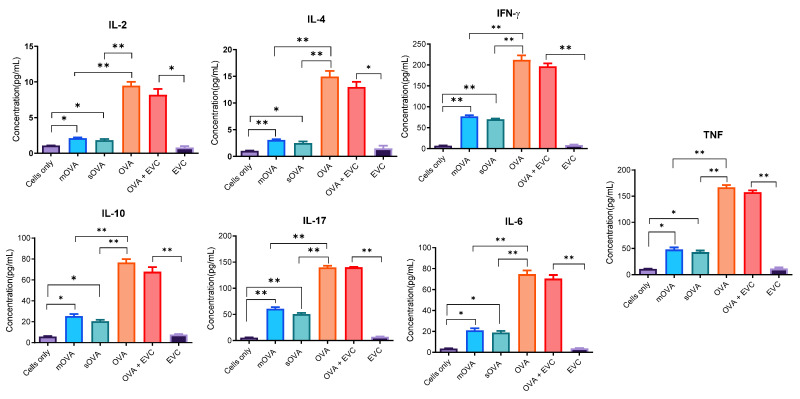
Cytokine expression of recovered cells following ex vivo stimulation. A single-cell suspension was prepared from the splenic, maxillary, axillary, and inguinal lymph nodes of OT-II mice for antigenic stimulation of either the whole fraction or the non-purified cells. After the injection of 2 × 10^6^ CFSE-labelled OT-II cells into the CD45.1-congenic mice, 5 × 10^6^ OVA-expressing *Leishmania* promastigotes and empty-vector-transformed promastigotes (EVC) were given s.c. to each mouse. Unchallenged OVA- (10 μg) and CFSE-labelled cells were used as a negative control. The time between the first day of cell injection and the challenge with the antigens was 24 h. After the challenge, infection was maintained for a week before the mice were culled for cell recovery. After a week, recovered cells were stimulated ex vivo with each antigen used for the challenge. Cells were further cultured in vitro for four days, and culture supernatants were analysed for their expression of cytokines using a cytokine bead array and flow cytometry. The cytokine concentrations are expressed in pg/mL. EVC stands for empty-vector control-transfected *Leishmania* parasites. *, *p* < 0.05; **, *p* < 0.01.

To confirm the in vivo and ex vivo activation of the OT-II cells recovered from the congenic mice, we analysed the cytokine secretion profile of OT-II cells recovered from the congenic mice and stimulated ex vivo. As shown in Figure 7, seven cytokines, namely IL-2, IL-4, IL-6, IL-10, IL-17, TNF, and IFN-γ, were produced by cells stimulated by the mOVA and sOVA. As expected, OT-II T cells recovered from mice challenged or immunised with the OVA protein followed by ex vivo stimulation with the same protein also showed a significant level of cytokine expression. On the other hand, cells that were not challenged with any of the antigens and those challenged with empty-vector-transfected *Leishmanial* parasites (EVC) showed a background-level expression of cytokines. Regarding cytokines, the concentrations found in the supernatants of cells stimulated with OVA-expressing parasites (mOVA and sOVA) were significantly higher (*p* < 0.05) than the amount of cytokines expressed by cells exposed to no stimulation (cells only) or empty-vector-transfected parasites (EVC) (Figure 7).

### 2.8. Stimulation and Proliferation Rate of Recovered Antigen-Experienced Cells

**Figure 8:**
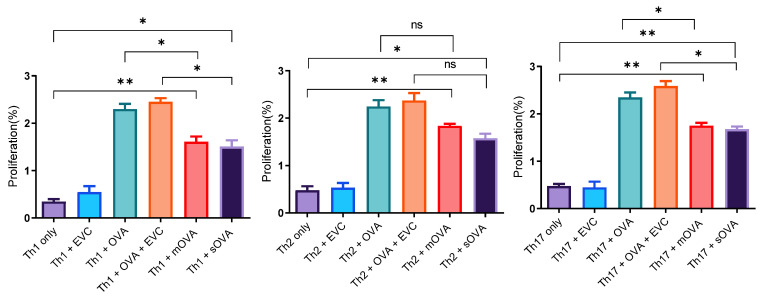
Proliferation of polarised OT-II cells following ex vivo stimulation. A single-cell suspension was prepared from the splenic, maxillary, axillary, and inguinal lymph nodes of OT-II mice for antigenic stimulation of either the whole fraction or the non-purified cells. After the injection of 2 × 10^6^ polarised OT-II cells into the CD45.1-congenic mice, 5 × 10^6^ OVA-expressing *Leishmania* promastigotes and empty-vector-transformed promastigotes (EVC) were given s.c. to each mouse. OVA (10 μg) and cells without the challenge were used as positive and negative controls, respectively. The time between the first day of cell injection and the challenge with the antigens was 24 h. After the in vivo challenge, infection was maintained for one week before the OT-II cells were recovered for ex vivo restimulation for 12 h. The proliferation rate was estimated using flow cytometry by dividing the total proliferated cells and anti-CD4 marker-positive T cells by the total CD4^+^ T cells recovered. EVC stands for empty-vector control-transformed *Leishmanial* parasites. *, *p* < 0.05; **, *p* < 0.01; ns, not significant.

In the previous section, naïve OT-II cells were stimulated with various forms of OVA, but these cells were not polarised prior to their adoptive transfer. In order to understand whether *L. major* affects the Th phenotype of OT-II cells and hence the degree of resilience of these cells, it was essential to polarise the OT-II cells prior to their adoptive transfer and demonstrate that these cells can be stimulated by OVA-expressing *L. major*. Therefore, naïve OT-II T cells were polarised in vitro into different Th1, Th2, and Th17 cells using polarising cytokines, antibodies to cytokines, and OVA peptide and differentiated into memory cells using IL-7. These cells were injected intravenously into recipient mice and after 24 h, each group of recipients was challenged subcutaneously with different antigens: OVA protein (20 μg/mL) as a positive control, 5 × 10^6^ empty-vector-transformed promastigotes mixed with the same concentration of OVA (20 μg/mL), 5 × 10^6^ OVA-expressing log-phase promastigotes, or the same quantity of empty-vector control-transfected *Leishmania* parasites (EVC). In addition, mice injected with each phenotype of polarised cells were left unchallenged to serve as a negative control. After a week, memory OT-II T cells were recovered from the spleens and lymph nodes of these congenic mice and further stimulated ex vivo with whichever antigen was used for the challenge and incubated for four days. In all cases (i.e., Th1, Th2, and Th17), the polarised OT-II T cells proliferated when stimulated with OVA (10 μg), mOVA-expressing parasites, or sOVA-expressing parasites (Figure 8). The results show that following ex vivo stimulation, the OVA protein induced a significantly higher amount of proliferation compared to both types of OVA-expressing parasites (Figure 8). Similarly, recovered cells of each phenotype showed a higher percentage of proliferation when stimulated with OVA-expressing *Leishmania* parasites compared to empty-vector-transfected *Leishmania* parasites and when cells were left without antigen stimulation (Figure 8). A marginal number of cells showed proliferation when recovered from mice challenged with the EVC treatment alone or from those that remained unchallenged by any antigen.

### 2.9. Cytokine Production by Recovered Th-Polarised Cells Following Stimulation with OVA-Expressing L. major

**Figure 9:**
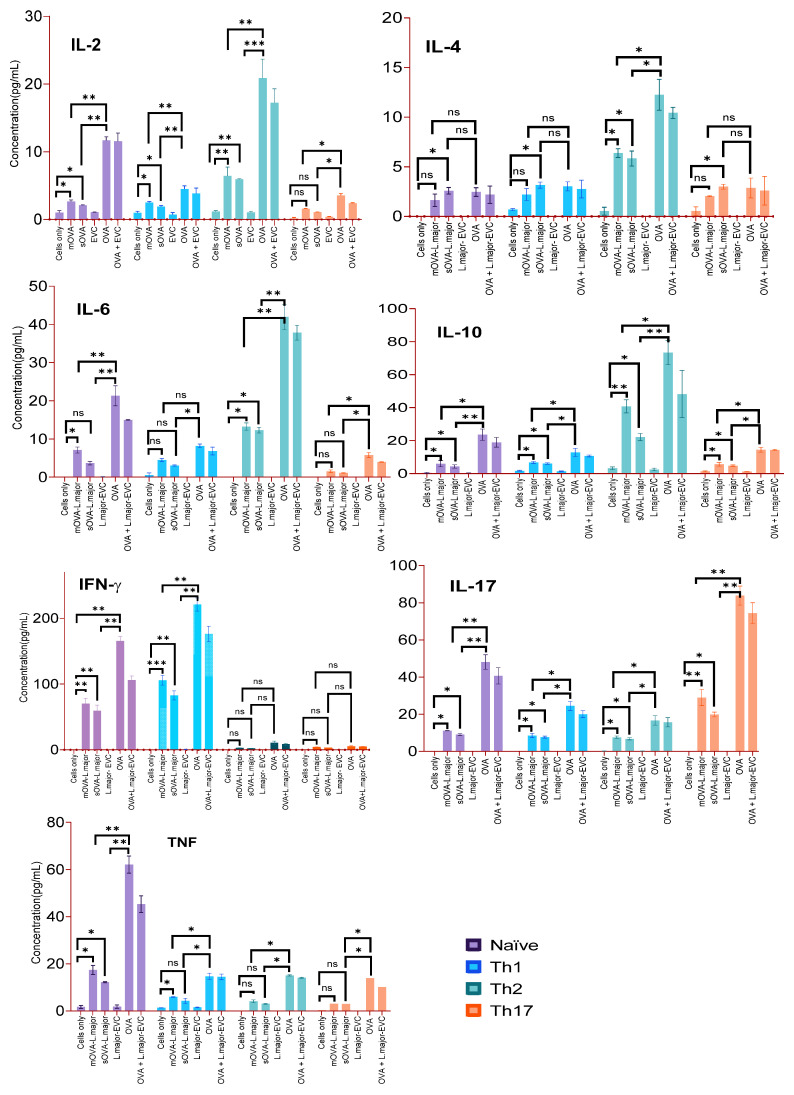
Detection of cytokine signatures from polarised OT-II T cells following antigen-specific ex vivo stimulation. A single-cell suspension was prepared from the splenic, maxillary, axillary, and inguinal lymph nodes of OT-II mice for antigenic stimulation of either the whole fraction or the non-purified cells. After the injection of 2 × 10^6^ Th phenotype-differentiated OT-II cells into the CD45.1-congenic mice, 5 × 10^6^ OVA-expressing *Leishmania* promastigotes and empty-vector-transformed promastigotes (EVC) were given s.c. to each mouse. OVA (10 μg) and cells without the challenge were used as a positive and negative control, respectively. The time between the first day of cell injection and the challenge with the antigens was 24 h. After the challenge, infection was maintained for a week before the mice were culled for cell recovery. After a week, recovered cells were stimulated ex vivo for 12 h with each antigen used for the challenge separately. Then, cells were further cultured in vitro for four days, and culture supernatants were analysed for their expression of cytokines using flow cytometry. *, *p* < 0.05; **, *p* < 0.01; ***, *p* < 0.001; ns, not significant.

After the in vitro polarisation of naïve OT-II T cells into Th1, Th2, and Th17 cells using polarising cytokines and OVA peptide (10 μg/mL), memory cells were produced using IL-7. Then, approximately 2 × 10^6^ naïve, Th1-, Th2-, and Th17-polarised OT-II T cells were injected intravenously into recipient mice and after 24 h, each group was challenged by subcutaneous injections with the following antigens: OVA protein (10 μg) as a positive control, 5 × 10^6^ parasites mixed with the same concentration of OVA, 5 × 10^6^ OVA-expressing log-phase promastigotes, or the same quantity of empty-vector-transformed *Leishmania* parasites (EVC). In addition, some mice were injected with each phenotype of polarised cells and left unchallenged to serve as a negative control. After one week, antigen-experienced cells were recovered from the spleens and lymph nodes of the congenic mice and further stimulated ex vivo with whichever antigen was used for the challenge and incubated for four days. Then, the cells were analysed for their production of cytokines, as shown in Figure 9. Different cytokines were produced at various concentrations. The results show that for the negative controls (i.e., cells only and EVC) there was either a non-detectable amount of cytokines or marginal amounts. Naïve cells stimulated with OVA or EVC + OVA produced each of the cytokines tested. Naïve cells stimulated with mOVA or sOVA produced less of each cytokine compared to when they were stimulated with OVA. Interestingly, the cytokine profiles of the differentiated OT-II cells, whether Th1, Th2, or Th17, showed that they retained their cytokine secretion profile when stimulated with OVA or EVC + OVA (Figure 9). Indeed, Th1 cells continued to produce IFN-γ, irrespective of the stimuli (Figure 9). Th2 cells continued to produce predominantly Th2 cytokines such as IL-2, IL-4, IL-6, and IL-10, irrespective of the stimuli (Figure 9). Similarly, Th17 cells produced IL-17 at a higher level than any of the other differentiated OT-II cells (Figure 9). Conversely, Th2 and Th17 cells produced very little IFN-γ, while Th1 cells produced very little Th2 or Th17 cytokines. Beside IL-17, the Th17 cells did not produce any other cytokines to any great extent. Thus, irrespective of the stimuli provided, the differentiated cells continued to produce the cytokines that they were previously differentiated to produce.

### 2.10. Measuring Immune Memory Resilience during L. major Exposure

**Figure 10:**
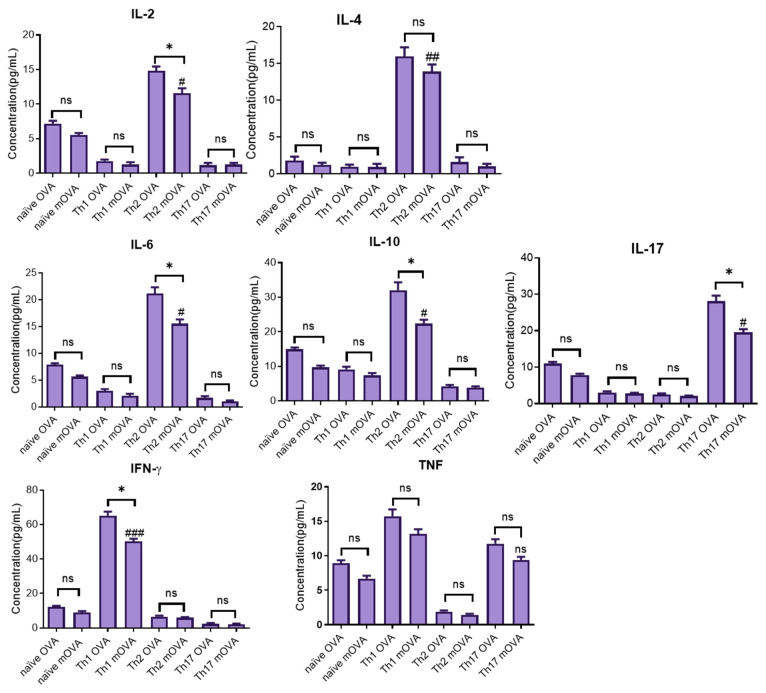
Cytokine signatures of antigen-experienced cells subjected to an in vivo challenge with OVA-transfected parasites or a cells-only control treatment following ex vivo stimulation with OVA protein. A single-cell suspension was prepared from the splenic, maxillary, axillary, and inguinal lymph nodes of OT-II mice for antigenic stimulation of either the whole fraction or non-purified cells. Then, naïve OT-II T cells were polarised into Th1/Th2/Th17 phenotypes. Then, a total of 2 × 10^6^ cells were transferred into recipient congenic mice (CD45.1, n = 6, iv) and, after 24 h, each mouse was challenged with 5 × 10^6^ log-phase promastigotes (mOVA-expressing *L. major*) and the OVA protein (20 μg OVA). Control mice received naïve undifferentiated OT-II T cells. After seven days, the recipient mice were terminated, and their antigen-experienced memory cells were processed and restimulated with 2 μg OVA. Finally, cytokine signatures were analysed in the culture supernatants. * or ^#^, *p* < 0.05; ^##^, *p* < 0.01; ^###^, *p* < 0.001; ns, not significant.

In this study, the immune memory resilience of antigen-experienced cells (i.e., their ability to withstand manipulation by pathogens) was measured in vivo by analysing their cytokines in response to challenges with *L. major.* The results showed that all antigen-experienced cells produced the same cytokine signatures when analysed using flow cytometry. As illustrated in Figure 10, following the injection of congenic mice with naïve unpolarised cells and Th1-, Th2-, and Th17-polarised cells, each group of mice was challenged either with OVA (10 μg) or mOVA-expressing *L. major*, and, finally, recovered unpolarised (naïve) and antigen-experienced cells were further cultured in vitro through stimulation with OVA (10 μg/mL). The memory cells were analysed to measure their cytokine signatures and the results showed that mice injected with naïve cells produced a lower level of cytokines (less than 10 pg/mL), whereas mice injected with the Th1 phenotype followed by a challenge with OVA or mOVA-expressing *L. major* produced cytokine signatures with concentrations of IFN-γ (65.2 pg/mL; 50.1 pg/mL) and TNF (15.8 pg/mL; 13.2 pg/mL), respectively. In addition, mice injected with Th2 phenotypes followed by a challenge with OVA or mOVA-expressing *L. major* produced IL-10 (32.1 pg/mL; 22.4 pg/mL) and IL-6 (21.2 pg/mL; 15.6 pg/mL), respectively. Similarly, mice injected with the Th17 phenotype followed by a challenge with OVA or mOVA-expressing *L. major* produced IL-17 (28.2 pg/mL; 19.6 pg/mL) and TNF (11.8 pg/mL; 9.4 pg/mL), respectively. The statistical association between the cytokines produced by the primed cells compared to those produced by the non-primed or naïve cells following exposure to antigen stimulation showed that Th1 cytokines (except TNF), Th2 cytokines, and Th17 cytokines were expressed at significantly higher levels compared to the levels expressed in the unprimed cells following antigenic stimulation with mOVA-expressing parasites. In comparison, the naïve OT-II T cells produced a minimum number of cytokines following exposure to OVA-expressing parasites and the OVA protein (Figure 10).

## 3. Discussion

The efficacy of experimental *Leishmania* vaccines is significantly influenced by innate immune cells. For example, neutrophils play a critical role in vaccine immunity due to their capacity to present antigens to naïve T cells. Skin-resident CD4+ memory T cells recruit inflammatory monocytes (iMOs) and contribute to controlling *L. major* infection by producing inducible nitric oxide synthase and nitric oxide. During vaccine immunity, the function of macrophages in enhancing a Th1 or Th2 response has been reported in *L. donovani* infections. In addition, the role of DCs as a source of IL-12 and IL-12’s role in the pathogenesis and induction of an efficacious vaccination response in *Leishmania* has also been reported [24]. Immunisation using centrin-deficient *L. mexicana* parasites (*LmexCen^−/−^)* following the generation of CD4+ T memory cells showed a protective immunity that could be maintained. These genetically manipulated parasites can potentially be used for vaccination and can provide protection against *L. mexicana* infection [25]. Changes in MHC-II/peptide affinity, antigen presentation, protease-susceptible sites, and intracellular expressions of pathogenic proteins during *Leishmania* infection can affect the reactivation of targeted antigen-specific T cells produced during priming, as well as dominant epitope selection. When *Leishmania*-infected macrophages provide antigens to memory T cells that are specific to that antigen, the T cells’ functional phenotype may shift, resulting in either inactivity or death. The peptides produced during infection may differ from and cross-react with the priming peptides, even though cells may be stimulated. The otherwise active T lymphocytes that are specific to antigens may be suppressed by such modified peptide ligands [26]. In this study, we hypothesised that long-lasting protective immunity in a host can be induced through the development of immune memory T cells and through the ability of these cells to induce appropriate secondary responses to specific pathogenic infections. This can be achieved through the generation of a resilient immune memory that can withstand the parasite’s manipulation process. Failure to produce such a resilient immune memory response through vaccination would allow the pathogen to manipulate the induced immune memory and hence circumvent the protection offered by the vaccine. Hence, as a matter of principle, it is critical to ascertain that induced polarised immune memory responses can withstand this manipulation (i.e., are resilient). This can best be assessed using the model developed here, where polarised immune memory T cells are generated and challenged in the context of the pathogen. With this aim, we generated *L. major* parasites that expressed two forms of OVA, namely membrane-associated OVA (mOVA) and soluble OVA (sOVA). We have shown that the expression of both forms of *L. major*-expressed OVA can stimulate OT-II cells in vitro and in vivo. These results are unexpected because previous studies have shown that while mOVA could stimulate OT-II cells, soluble (or cytosolic) sOVA could only do so at a higher dose [26]. Indeed, Prickett et al., 2006 [27], used *L. major* hydrophilic acylated surface protein B for the generation of OVA-transgenic parasites expressing the antigen either in the cytosol or in the membrane. Only the membrane-associated OVA was recognised and led to the activation of CD4^+^ T cells at low parasite doses, but both mOVA- and sOVA-producing parasites could stimulate immune recognition at higher concentrations. Such studies support the importance of antigen localisation during the recognition of antigens by T cells [28]. However, in our experiments, we used higher parasite doses, which could account for the observed stimulation of OT-II cells with sOVA at a similar level to mOVA. In addition, Prickett et al. used DO11.10 TCR Tg mice [27], while we used OT-II TCR-transgenic animals, and the amount of accessible OVA required to stimulate these two different T cells might be different. Our results are compatible with those of Kaye et al., 1993 [29], suggesting that *L. major* strains expressing OVA and β-gal in the phagosome as a soluble antigen are efficient at inducing CD4^+^ T cell activation. Nevertheless, in later experiments, when the number of available animals was limited and we had to choose only one type of parasite, we chose mOVA.

Although the level of cytokines produced was generally much smaller than in the positive controls containing the OVA protein, every cytokine measured was produced following stimulation with both sOVA- and mOVA-expressing parasites. The relatively low level of cytokine production was not due to an inhibitory or toxic effect of the parasites on the OT-II cells, as control parasites supplemented with soluble OVA protein secreted cytokines at a similar rate to the OVA protein-stimulated OT-II cells. Thus, it is likely that the relatively low level of cytokine secretion is due to the relatively low amount of OVA protein produced by the parasite and/or the way that the parasite-produced OVA is processed within the APCs. Indeed, it has been suggested that the antigen processing of *L. major*-produced OVA might be different to that of the soluble OVA protein, as it was found that phagolysosome-targeted OVA expressed in *L. major* was able to be presented by macrophages for a period of 24 h, unlike these same macrophages pulsed with soluble OVA [28]. Therefore, if indeed OVA produced by the parasite can be presented for a prolonged period of time, it is likely that the concentration of peptides associated with the MHC II molecules on the surface of these cells at any given time might be lower than in the case of soluble OVA present in the surrounding environment.

We have also demonstrated the use of OT-II cells that were readily polarised in vitro, and these polarised OT-II cells behaved as expected and could be restimulated not only by OVA but importantly also by *L. major* expressing both mOVA and sOVA. This opened the door to in vivo experiments using OT-II cells transferred to recipient mice. This is important as intracellular parasites such as *Leishmania*, living inside the harsh environment of phagocytes, have developed strategies and physiological adaptations to escape the immune system of the host. Some of these mechanisms involve the manipulation of the host’s immune response to achieve, for example, inhibition of the signalling pathways of the host’s macrophages, leading to parasite survival [30]. Different in vitro studies have confirmed that IL-10, one of the anti-inflammatory cytokines, is produced by macrophages parasitised with *Leishmania* through interaction with the Fcγ receptor [31]. The production of IL-10 leads into the suppression of the microbicidal nature, which involves nitric oxide, and the production of cytokines such as TNF, IL-1, and IL-12. The expression of costimulatory molecules such as B7-1/2 has also been found to be involved in the process of inhibiting the microbicidal activities of macrophages [32]. The clinical implication of IL-10 expression has been studied in vivo and the results indicate that transgenic mice expressing IL-10 fail to control parasites [33]. However, the findings from our study demonstrate that in the presence of Th-polarised cells, the production of IL-10 is not associated with the progression of *Leishmania* infection at a higher rate, as the parasitic load was found to be significantly reduced in mice injected with either Th1, Th2, or Th17 cells followed by a challenge with OVA-expressing parasites and a parasite control. This was unexpected as Th2 cells produce significantly more IL-10 compared to Th1 and Th17 cells and hence we expected that, based on previous studies [34], the presence of IL-10 would have promoted the parasite’s development.

The efficacy of experimental *Leishmania* vaccines is significantly influenced by innate immune cells. For example, neutrophils, due to their antigen-presenting capacity in naïve T cells, play a critical role in vaccine immunity. Skin-resident CD4+ memory T cells recruit inflammatory monocytes (iMOs) and contribute to controlling *L. major* infection by producing inducible nitric oxide synthase and nitric oxide. The role of DCs as a source of IL-12 and IL-12’s role in the pathogenesis and potentiation of an efficacious vaccine response in *Leishmania* have also been reported [24].

Several experimental vaccines against *Leishmania* have been generated to halt the skin-associated and visceral forms of the disease, which are both contracted through the bite of an infected sand fly. Following a needle challenge, immunisation with autoclaved *L. major* (ALM) given with the Th1 adjuvant TLR9 agonist CpG oligodeoxynucleotides has been found to significantly protect against infection in resistant C57BL/6 mice. In a study in resistant C57BL/6 mice, the immunity produced by KSAC or L110f immunisation with GLA-SE after a challenge with L. major induced by a needle or an infected sand-fly bite was investigated. Since a sand-fly bite is expected to induce neutrophil recruitment, the influence of KSAC vaccination on the strength and Th1/Th2 nature of subsequent immune responses to *L. major* transmitted via sand-fly bite in these two mouse strains is likely to be in addition to any influence of neutrophils on the expression of this immunity [35]. In our study, after challenging in vitro-polarised memory T cells with OVA-expressing *L. major* parasites in vivo, the degree of immune memory resilience was measured. Naïve OT-II T cells were polarised into Th1, Th2, and Th17 cells in vitro and these cells’ phenotypes were confirmed by analysing their cytokine signatures. These cells were adoptively transferred to recipient mice, and they retained their cytokine secretion profiles when restimulated in vivo and analysed in vitro. This is a key finding as it suggests that in vitro-polarised OT-II cells can maintain their Th profile in vitro when subjected to *L. major* expressing OVA. This is extremely important for vaccine development as it suggests that if it was possible to vaccinate an individual in such a way as to generate immune memory cells similar to these produced in vitro, one should be able to protect them from infection. Adoptively transferring in vitro-differentiated Th1 cells specific for the *Leishmania*-specific CD44^+^PEPCK tetramer into recipient mice showed an immediate presence of circulating CD4^+^ T helper 1 effector cells (Th1_EFF_). These are critical for preventing *L*. *major*-mediated immunomodulation [36]. Experimental studies have shown that understanding the link between immune memory and protection against a parasite is very important as it has implications for efficacious vaccination. In addition, memory T cells are reported to be protective only if they generate circulating Teff cells before a challenge [37]. Two distinct populations of CD4(+) T cells, namely short-lived pathogen-dependent Teff cells and long-lived pathogen-independent Tcm cells, define immunity to *L. major* [38].

Skin-graft transplantation of *Leishmania*-specific CD4+ T memory cells into naïve mice produces IFN-γ, and the cells remain resident in the skin. They recruit circulating T cells to the skin in a CXCR3-dependent manner and control the parasite infection, and this allows us to draw the conclusion that optimal protective immunity to this parasite and effective vaccination depends on generating both circulating and skin-resident memory T cells [39]. Tissue-resident memory CD4^+^ T cells provide protection after a parasite challenge and this requires the recruitment and activation of inflammatory monocytes by producing reactive oxygen species and NO, which plays a critical role in immunity to cutaneous leishmaniasis [40]. After the resolution of primary infection, short-lived Teff cells, long-lived Tcm cells, and skin-resident memory T cells retain immunity to secondary infection. Although parasitic infection leads to the generation of protective immunity, vaccine efficacy depends on generating memory T cells that are sustained in the absence infection [41].

Indeed, induced protective immunity can be achieved when memory T cells develop the ability to retain the production of their cytokine signatures in the face of an immunomodulatory pathogen. To this end, a resilient immune memory was detected in vivo after the adoptive transferral of antigen-experienced cells into recipient mice followed by a challenge with *L. major*. Th1-polarised memory T cells showed the strongest proliferation and produced a significant amount of IFN-γ following challenges with OVA protein and OVA-expressing *L. major*. In addition, mice that received Th2-polarised memory T cells showed Th2 proliferation and produced a significant amount of IL-2, IL-4, IL-6, and IL-10 following challenges with OVA protein and OVA-expressing *L. major*. Similarly, mice that received Th17-polarised memory T cells showed OT-II cell proliferation and produced a significant amount of IL-17 following challenges with OVA protein and OVA-expressing *L. major*. However, due to several technical issues, we used a non-physiological number of parasites and T cells, and a non-physiological route of infection. One of the limitations of this study is the physiological number of parasites used. In this study, due to technical issues related with transfection efficacy and optimising the expression level of OVA protein by the parasites and inducing OVA-mediated T cell activation, a relatively high number of parasites (5 × 10^6^) was used compared to the number of parasites used in previously reported studies. The route of parasite infection is an important parameter to consider in different experimental models of *Leishmania* parasites. Although the most preferred route of parasite injection is intradermal to mimic natural infection and study immune responses, evidence from a previous study has shown that the subcutaneous route induces significantly more protective immunity against L. major, lowers the parasite load, allows rapid lesion resolution, and lowers IL-production [42]. However, we also recommend the intradermal route for the application of OVA-transgenic parasite models for future immune modulation studies. In addition, another limitation of this study is related to the non-optimal number of antigen-experienced cells used following the use of non-purified OT-II T cells from the spleen and lymph nodes. In this study, we also used empty-vector-transfected parasites as a negative control; however, in order to explicitly see if there is an immune response induced by the studied parasite, including a wild-type group is recommended for future studies.

## 4. Materials and Methods

### 4.1. Experimental Mice

Six- to eight-week-old female OT-II mice and CD45.1-congenic mice were used in the present study as sources of naïve OT-II T cells and for the adoptive transfer of polarised antigen-experienced cells, respectively, and were purchased from the Walter and Eliza Hall Institute of Medical Research, Melbourne, Australia. All mouse-related research projects require approval from the University of Melbourne’s Animal Ethical Committee (Ethics ID: 1814548.1). OT-II mice are OVA-specific, MHC class II restricted αβ TCR-transgenic mice. These OT-II-transgenic mice express the mouse alpha-chain and beta-chain T cell receptor that pairs with the CD4 co-receptor and is specific for chicken ovalbumin 323–339 peptide in the context of I-Ab (CD4 co-receptor interaction with MHC class II).

### 4.2. Leishmania Major Culture

To produce transfected parasites, the *Leishmania major* strain MHOM/SU/73/5-ASKH was employed as the wild type and axenic promastigotes were routinely cultivated in SDM-79 media supplemented with 10% FCS at 27 °C. Parasite cells were cultivated up to mid-log phase and extracted using centrifugation (2000× *g*, 10 min) for long-term storage. At 10^8^ cells/mL, parasites were resuspended in a mixture of 90% FCS and 10% DMSO. Each cryotube contained about 2 × 10^7^ cells, which were progressively frozen at −80 °C overnight before being transferred to liquid nitrogen.

### 4.3. Cloning of the OVA Gene into L. major Expression Vector pIR1SAT

The gene in sOVA that codes for full-length OVA was digested to obtain a size of 1158 bp using SmaI and XhoI. The Klenow enzyme was used to produce blunt ends. In order to prevent vector re-ligation, the plasmid vector pIR1SAT [43,44], also known as pSAT, was linearised using SmaI and treated with 1 U of shrimp alkaline phosphatase per μg of DNA. Plasmids were obtained from ampicillin-resistant clones and screened by restriction digestion with EcoRV, which produced a 771 bp DNA fragment via insert ligation into the vector with the proper orientation. One EcoRV restriction site on the vector and one on the insert’s surface made it possible to screen for ampicillin-resistant clones that were correctly put into the vector. Additionally, transformants were identified by colony PCR utilising forward and reverse primers created on the vector backbone and seen with agarose gel electrophoresis in ampicillin-resistant clones. For *L. major* transfection, pSAT and pSAT-OVA were digested with SwaI and the targeted fragment was extracted via gel extraction [45].

### 4.4. Genetic Manipulation of L. major

SwaI was used to digest pSAT and pSAT-derived plasmids to extract the desired area for transfection. With a few adjustments, a fragment of the right size that corresponded to the gene of interest and the targeting region for the ssu gene was electroporated into *L. major* using gel purification [46,47]. Centrifugation (1000× *g*, 10 min) was used to collect mid-log-phase parasites (0.8 × 10^7^ cells/mL). After being washed in 20–30 mL of cold electroporation buffer (1000× *g*, 10 min), a total of 4 × 10^7^ cells per transfection were resuspended in 500 μL of cold electroporation buffer. Cuvettes were filled with the prepared cell suspension, and two 1.7 kV, 25 F capacitance electrophoretic pulses were administered. *L. major* was transfected with either 5 μg of DNA or no DNA (control). Recovery of the parasites took place in 10 mL of SDM-79 media supplemented with 100 μg/mL of penicillin/streptomycin for 24 h at 27 °C. The selection pressure for the pSAT plasmids was then maintained by diluting the parasite cultures with 15 mL of SDM-79 medium and nourseothricin (Werner BioAgents, Jena, Germany, 110 μg/mL).

### 4.5. Confirming OVA Expression by L. major

Promastigotes were suspended in 200 µL of lysis buffer (Thermo Fisher Scientific, Waltham, MA, USA) once they reached the log phase, and 2 × 10^6^ live promastigotes were then heated to 65 °C for 10 min. Using light microscopy, the parasite lysate was centrifuged at 9400× *g* for 2 min to ensure parasite death. When complete breakdown was attained, the parasite homogenates were subjected to ultrasonic-dependent electronic pulses once more, utilising the Microson^TM^ ultrasonic cell disruptor. The supernatants were then purified by passing them through a 0.2 µm filter following high-speed centrifugation. To confirm the above-described protein expression, the parasite lysates were separated using SDS-PAGE and then submitted to Western blotting using anti-OVA antibodies.

### 4.6. Lymphocyte Preparation

Maxillary, inguinal, and splenic lymph nodes of the OT-II and CD45.1 mice were combined in RPMI-1640 (Thermo Fisher Scientific, Waltham, MA, USA). A single-cell suspension was prepared from the collected lymph nodes for antigenic stimulation of either the whole fraction or the non-purified cells. For all of the assays, CD45.1 and CD45.2 cells were stained after counting their proportion using flow cytometry from the whole extracted cells. Forceps were used to crush the tissues, and a 70 µm nylon cell strainer was used for the filtering process. The cells were centrifuged at 276× *g*, for six minutes. After aspirating the supernatants, distilled water was used to lyse the red-blood-cell pellets for 9 s, after which 10% of 10X PBS was added to stop the lysis. This cell suspension was centrifuged at 276× *g*, for six minutes. The cells were resuspended in new complete RPMI-1640 media supplemented with 2 mM L-glutamine, 100 U/mL penicillin, 100 g/mL streptomycin, 10% *v*/*v* heat-inactivated FCS, and 50 μM 2-Mercaptoethanol after the supernatants had been removed. Cell concentrations were calculated where necessary using a haemocytometer.

### 4.7. In Vitro Polarisation of OT-II T Cells

OT-II cells were polarised in vitro as previously described [48]. The following differentiation cytokines were used to polarise the naïve OT-II cells into three lineages: 2 × 10^5^ cells per well were cultured for 4 days and polarised into Th1 (using 10 μg/mL OVA peptide and 10 ng/mL of IL-2), Th2 (using 10 μg OVA peptide and 10 ng/mL of IL-2, 10 ng/mL of IL-4, and 10 μg/mL of anti-IFN-γ), and Th17 (using 10 μg/mL OVA peptide, 10 μg/mL of anti-IL-4, 10 μg/mL of anti-IFN-γ antibodies, 5 ng/mL of anti-TGF β, and 20 ng/mL of IL-6). Naïve cells were activated with 10 μg/mL of OVA peptide. To create memory cells [49], cells were further stimulated for two days with 4.5 ng/mL of IL-7 (Pepro Tech, Rocky Hill, NJ, USA). Th-polarised cells were stimulated with OVA peptide (10 μg/mL) for 72 h using 10^5^ cells per well. Using flow cytometry, the level of polarisation was assessed based on the cytokine profiles of the differentiated cells.

### 4.8. Adoptive Transferral of OT-II T Cells to Congenic Mice and Parasite Injection

According to the manufacturer’s instructions, cells were stained with carboxyfluorescein-diacetate-succinimidyl-ester (CFSE; Bio Legend, San Diego, CA, USA). Following this, 2 × 10^6^ CD45.2-expressing OT-II T cells were adoptively transplanted into CD45.1-congenic mice. As a route of infection, we used the subcutaneous method of administration, injecting subcutaneously (s.c.) under the tail base of the recipient mice. Each animal received (s.c.) an injection of 5 × 10^6^ empty-vector control-transformed promastigotes (EVC) and OVA-expressing *Leishmania* promastigotes. As a negative control, cells labelled with CFSE but not challenged with OVA (20 ng/mL) were employed. As a negative control, unchallenged OT-II T cells were employed. Different antigens were administered to the mice after 24 h. Mice were killed after 7 days in order to collect OT-II T cells from the spleen for analysis.

### 4.9. T Cell Proliferation Assay

Cells were extracted from the splenic, maxillary, axillary, and inguinal lymph nodes of the OT-II mice using complete RPMI-1640 media [50]. The tissue was run through a 70 µm nylon cell strainer to create single-cell suspensions. RBCs were lysed according to the previously mentioned method. Following this, cells were labelled with CFSE in accordance with the manufacturer’s instructions. A total of 2 × 10^5^ CFSE-labelled cells per well were co-cultured for 4–5 days with OVA-transgenic or wild-type log-phase parasite promastigotes. OT-II cells were collected and washed with FACS buffer (10% FCS in PBS) after four days of culture. Following an Fc γR-surface block (anti-Fc γR, clone 2.4G2-16, WEHI Monoclonal Facility, Parkville, Australia), cells were stained with PE-anti-mouse CD4 (Bio Legend, San Diego, CA, USA) for proliferation analysis. Prior to data collection, using a BD FACSVerseTM flow cytometer (BD Bioscience, Franklin Lakes, NJ, USA), 7-amino-actinomycin D (1 µg/mL; Sigma Aldrich, Steinheim, Germany) was added to the cell suspension in order to remove dead cells. Similar to this, adoptively transferred antigen-experienced cells were collected from recipient mice’s spleens after a week, and their proliferation was examined following an ex vivo parasite restimulation.

### 4.10. Cytokine Assay

A mouse Th1, Th2, and Th17 cytometric bead array (BD^TM^) was used to measure the cytokine production in cell culture supernatants from the T cell proliferation tests. The detection limits for each cytokine were as follows: IL-2 (0.1 pg/mL), IL-4 (0.03 pg/mL), IL-6 (1.4 pg/mL), IFN-γ (0.5 pg/mL), TNF (0.9 pg/mL), IL-17A (0.8 pg/mL), and IL-10 (16.8 pg/mL), according to the manufacturer.

### 4.11. Statistical Analyses

The single-cell analysis programme Flowjo version 10 (Ashland, OR, USA) was used to compute and analyse the data. A one-way ANOVA was used for all statistical analyses using GraphPad Prism version 7.00 for Windows (GraphPad Software, La Jolla, CA, USA). The confidence level was maintained at 95% for all analyses, and a significant threshold of *p* ≤ 0.05 was employed.

## 5. Conclusions

In vitro-polarised Th1, Th2, and Th17 phenotypes remain resilient in vivo in the face of pathogen-mediated manipulation. The strongest immune memory response was observed in response to challenges with the OVA protein or OVA-expressing *L. major* parasites as measured by the proliferation rate of recovered Th1 memory cells and the amount of IFN-γ production. This could reflect the fact that Th1 memory T cells have developed an intrinsic nature to strongly respond to OVA antigens in the face of immunomanipulative pathogens because the recall response of the Th1 memory cells to OVA_323–339_ was higher in terms of their proliferative response and overall cytokine production, as confirmed by the data showing that mice who received Th1-polarised memory T cells produced higher quantities of IFN-γ, but not other cytokines. In addition, our findings regarding the effect of antigen localisation on measuring the strength of T cell responses also showed that mice that received Th1-, Th2-, and Th17-polarised cells and were challenged with mOVA-expressing *L. major* revealed relatively higher proliferative responses in vitro and in vivo compared to the mice that received the same group of antigen-experienced cells but were challenged with sOVA-expressing *L. major*. This was confirmed by measuring the proliferation of recovered memory T cells or by measuring the amount of cytokines they produced following restimulation with the OVA protein. The results of this study provide evidence showing that it is possible to induce resilient immune memory responses to *L. major* antigens (in this case, against OVA expressed by the studied parasite, but in the future, against other more relevant antigens as well) that can withstand manipulation by the parasite and hence offer protection even in the face of such manipulation.

## Data Availability

The corresponding author can provide the datasets used and/or analysed during the current work upon request.

## References

[1] Pearson R.D., Wheeler D.A., Harrison L.H., Kay H.D. (1983). The immunobiology of leishmaniasis. Rev. Infect. Dis..

[2] Scott P., Novais F. (2016). Cutaneous leishmaniasis: Immune responses in protection and pathogenesis. Nat. Rev. Immunol..

[3] Liew F.Y., Hale C., Howard J.G. (1982). Immunologic regulation of experimental cutaneous leishmaniasis. V. Characterization of effector and specific suppressor T cells. J. Immunol..

[4] Carvalho E.M., Badaró R., Reed S.G., Jones T.C., Johnson W.D. (1985). Absence of gamma interferon and interleukin 2 production during active visceral leishmaniasis. J. Clin. Investig..

[5] Murray H.W., Rubin B.Y., Carriero S., Acosta A.M. (1984). Reversible defect in antigen-induced lymphokine and gamma-interferon generation in cutaneous leishmaniasis. J. Immunol..

[6] Sadick M.D., Locksley R.M., Tubbs C., Raff H.V. (1986). Murine cutaneous leishmaniasis: Resistance correlates with the capacity to generate interferon-gamma in response to *Leishmania* antigens in vitro. J. Immunol..

[7] Nathan C.F., Murray H.W., Wiebe M.E., Rubin B.Y. (1983). Identification of interferon-gamma as the lymphokine that activates human macrophage oxidative metabolism and antimicrobial activity. J. Exp. Med..

[8] Murray H.W., Rubin B.Y., Rothermel C.D. (1983). Killing of intracellular *Leishmania donovani* by lymphokine-stimulated human mononuclear phagocytes. Evidence that interferon-gamma is the activating lymphokine. J. Clin. Investig..

[9] Crawford R.M., Finbloom D.S., Ohara J., Paul W.E., Meltzer M.S. (1987). B cell stimulatory factor-1 (interleukin 4) activates macrophages for increased tumoricidal activity and expression of Ia antigens. J. Immunol..

[10] Blackwell J.M., Ezekowitz R.A., Roberts M.B., Channon J.Y., Sim R.B., Gordon S. (1985). Macrophage complement and lectin-like receptors bind *Leishmania* in the absence of serum. J. Exp. Med..

[11] Mosser D.M., Edelson P.J. (1985). The mouse macrophage receptor for C3bi (CR3) is a major mechanism in the phagocytosis of *Leishmania* promastigotes. J. Immunol..

[12] Reiner S.L., Locksley R.M. (1995). The regulation of immunity to *Leishmania* major. Annu. Rev. Immunol..

[13] Sacks D., Noben-Trauth N. (2002). The immunology of susceptibility and resistance to *Leishmania major* in mice. Nat. Rev. Immunol..

[14] Scott P., Artis D., Uzonna J., Zaph C. (2004). The development of effector and memory T cells in cutaneous leishmaniasis: The implications for vaccine development. Immunol. Rev..

[15] Mond J.J., Carman J., Sarma C., Ohara J., Finkelman F.D. (1986). Interferon-gamma suppresses B cell stimulation factor (BSF-1) induction of class II MHC determinants on B cells. J. Immunol..

[16] Reynolds D.S., Boom W.H., Abbas A.K. (1987). Inhibition of B lymphocyte activation by interferon-gamma. J. Immunol..

[17] Gajewski T.F., Joyce J., Fitch F.W. (1989). Antiproliferative effect of IFN-gamma in immune regulation. III. Differential selection of TH1 and TH2 murine helper T lymphocyte clones using recombinant IL-2 and recombinant IFN-gamma. J. Immunol..

[18] Lehn M., Weiser W.Y., Engelhorn S., Gillis S., Remold H.G. (1989). IL-4 inhibits H_2_O_2_ production and antileishmanial capacity of human cultured monocytes mediated by IFN-gamma. J. Immunol..

[19] Pagán A.J., Peters N.C., Debrabant A., Ribeiro-Gomes F., Pepper M., Karp C.L., Jenkins M.K., Sacks D.L. (2013). Tracking antigen-specific CD4+ T cells throughout the course of chronic *Leishmania major* infection in resistant mice. Eur. J. Immunol..

[20] Heinzel F.P., Sadick M.D., Holaday B.J., Coffman R.L., Locksley R.M. (1989). Reciprocal expression of interferon gamma or interleukin 4 during the resolution or progression of murine leishmaniasis. Evidence for expansion of distinct helper T cell subsets. J. Exp. Med..

[21] Scott P. (1989). The role of TH1 and TH2 cells in experimental cutaneous leishmaniasis. Exp. Parasitol..

[22] Romano A., Carneiro M.B.H., Doria N.A., Roma E.H., Ribeiro-Gomes F.L., Inbar E., Lee S.H., Mendez J., Paun A., Sacks D.L. (2017). Divergent roles for Ly6C+CCR2+CX3CR1+ inflammatory monocytes during primary or secondary infection of the skin with the intra-phagosomal pathogen *Leishmania major*. PLoS Pathog..

[23] Peters N.C., Pagán A.J., Lawyer P.G., Hand T.W., Henrique Roma E., Stamper L.W., Romano A., Sacks D.L. (2014). Chronic parasitic infection maintains high frequencies of short-lived Ly6C+CD4+ effector T cells that are required for protection against re-infection. PLoS Pathog..

[24] Volpedo G., Pacheco-Fernandez T., Bhattacharya P., Oljuskin T., Dey R., Gannavaram S., Satoskar A.R., Nakhasi H.L. (2021). Determinants of Innate Immunity in Visceral Leishmaniasis and Their Implication in Vaccine Development. Front. Immunol..

[25] Volpedo G., Pacheco-Fernandez T., Holcomb E.A., Zhang W.W., Lypaczewski P., Cox B., Fultz R., Mishan C., Verma C., Huston R.H. (2022). Centrin-deficient Leishmania mexicana confers protection against New World cutaneous leishmaniasis. NPJ Vaccines.

[26] Zutshi S., Kumar S., Chauhan P., Bansode Y., Nair A., Roy S., Sarkar A., Saha B. (2019). Anti-Leishmanial Vaccines: Assumptions, Approaches, and Annulments. Vaccines.

[27] Prickett S., Gray P.M., Colpitts S.L., Scott P., Kaye P.M., Smith D.F. (2006). In vivo recognition of ovalbumin expressed by transgenic *Leishmania* is determined by its subcellular localization. J. Immunol..

[28] Beattie L., Evans K.J., Kaye P.M., Smith D.F. (2008). Transgenic *Leishmania* and the immune response to infection. Parasite Immunol..

[29] Kaye P.M., Coburn C., McCrossan M., Beverley S.M. (1993). Antigens targeted to the *Leishmania* phagolysosome are processed for CD4+ T cell recognition. Eur. J. Immunol..

[30] Olivier M., Gregory D.J., Forget G. (2005). Subversion mechanisms by which Leishmania parasites can escape the host immune response: A signaling point of view. Clin. Microbiol. Rev..

[31] Sutterwala F.S., Noel G.J., Salgame P., Mosser D.M. (1998). Reversal of proinflammatory responses by ligating the macrophage Fcgamma receptor type I. J. Exp. Med..

[32] Cunningham A.C. (2002). Parasitic adaptive mechanisms in infection by leishmania. Exp. Mol. Pathol..

[33] Kane M.M., Mosser D.M. (2000). *Leishmania* parasites and their ploys to disrupt macrophage activation. Curr. Opin. Hematol..

[34] Assreuy J., Cunha F.Q., Epperlein M., Noronha-Dutra A., O’Donnell C.A., Liew F.Y., Moncada S. (1994). Production of nitric oxide and superoxide by activated macrophages and killing of *Leishmania major*. Eur. J. Immunol..

[35] Peters N.C., Bertholet S., Lawyer P.G., Charmoy M., Romano A., Ribeiro-Gomes F.L., Stamper L.W., Sacks D.L. (2012). Evaluation of recombinant *Leishmania* polyprotein plus glucopyranosyl lipid A stable emulsion vaccines against sand fly transmitted *Leishmania major* in C57BL/6 mice. J. Immunol..

[36] Hohman L.S., Mou Z., Carneiro M.B., Ferland G., Kratofil R.M., Kubes P., Uzonna J.E., Peters N.C. (2021). Protective CD4+ Th1 cell-mediated immunity is reliant upon execution of effector function prior to the establishment of the pathogen niche. PLoS Pathog..

[37] Hohman L.S., Peters N.C. (2019). CD4+ T Cell-Mediated Immunity against the Phagosomal Pathogen *Leishmania*: Implications for Vaccination. Trends Parasitol..

[38] Zaph C., Uzonna J., Beverley S.M., Scott P. (2004). Central memory T cells mediate long-term immunity to *Leishmania major* in the absence of persistent parasites. Nat. Med. Oct..

[39] Glennie N.D., Yeramilli V.A., Beiting D.P., Volk S.W., Weaver C.T., Scott P. (2015). Skin-resident memory CD4+ T cells enhance protection against *Leishmania major* infection. J. Exp. Med. Aug..

[40] Glennie N.D., Volk S.W., Scott P. (2017). Skin-resident CD4+ T cells protect against *Leishmania major* by recruiting and activating inflammatory monocytes. PLoS Pathog..

[41] Glennie N.D., Scott P. (2016). Memory T cells in cutaneous leishmaniasis. Cell Immunol. Nov..

[42] Mahmoudzadeh-Niknam H., Khalili G., Abrishami F., Najafy A., Khaze V. (2013). The route of Leishmania tropica infection determines disease outcome and protection against *Leishmania major* in BALB/c mice. Korean J. Parasitol..

[43] Robinson K.A., Beverley S.M. (2003). Improvements in transfection efficiency and tests of RNA interference (RNAi) approaches in the protozoan parasite *Leishmania*. Mol. Biochem. Parasitol..

[44] Capul A.A., Barron T., Dobson D.E., Turco S.J., Beverley S.M. (2007). Two functionally divergent UDP-Gal nucleotide sugar transporters participate in phosphoglycan synthesis in *Leishmania major*. J. Biol. Chem..

[45] Mandal S., Maharjan M., Ganguly S., Chatterjee M., Singh S., Buckner F.S., Madhubala R. (2009). High-throughput screening of amastigotes of *Leishmania donovani* clinical isolates against drugs using a colorimetric beta-lactamase assay. Indian J. Exp. Biol..

[46] Vandenhoff M.J.B., Moorman A.F.M., Lamers W.H. (1992). Electroporation in Intracellular Buffer Increases Cell-Survival. Nucleic Acids Res..

[47] Bifeld E., Chrobak M., Zander D., Schleicher U., Schönian G., Clos J. (2015). Geographical sequence variation in the *Leishmania major* virulence factor P46. Infect. Genet. Evol..

[48] Tedla M.G., Every A.L., Scheerlinck J.Y. (2022). Measuring the Manipulation of T Helper Immune Responses by *Schistosoma mansoni*. Int. J. Mol. Sci..

[49] Kondrack R.M., Harbertson J., Tan J.T., McBreen M.E., Surh C.D., Bradley L.M. (2003). Interleukin 7 regulates the survival and generation of memory CD4 cells. J. Exp. Med..

[50] Tedla M.G., Nahar M.F., Hagen J., Every A.L., Scheerlinck J.-P.Y. (2019). Recognition of *Schistosoma mansoni* egg-expressed ovalbumin by T cell receptor transgenic mice. Exp. Parasitol..

